# Activin Signaling in Microsatellite Stable Colon Cancers Is Disrupted by a Combination of Genetic and Epigenetic Mechanisms

**DOI:** 10.1371/journal.pone.0008308

**Published:** 2009-12-14

**Authors:** Barbara Jung, Jessica Gomez, Eddy Chau, Jennifer Cabral, Jeffrey K. Lee, Aimee Anselm, Przemyslaw Slowik, Deena Ream-Robinson, Karen Messer, Judith Sporn, Sung K. Shin, C. Richard Boland, Ajay Goel, John M. Carethers

**Affiliations:** 1 Department of Medicine, University of California San Diego, La Jolla, California, United States of America; 2 Moores Cancer Center, University of California San Diego, La Jolla, California, United States of America; 3 Veterans Affairs San Diego Healthcare System, San Diego, California, United States of America; 4 Department of Medicine, Baylor University Medical Center, Dallas, Texas, United States of America; The University of Hong Kong, Hong Kong

## Abstract

**Background:**

Activin receptor 2 (*ACVR2*) is commonly mutated in microsatellite unstable (MSI) colon cancers, leading to protein loss, signaling disruption, and larger tumors. Here, we examined activin signaling disruption in microsatellite stable (MSS) colon cancers.

**Methods:**

Fifty-one population-based MSS colon cancers were assessed for ACVR1, ACVR2 and pSMAD2 protein. Consensus mutation-prone portions of *ACVR2* were sequenced in primary cancers and all exons in colon cancer cell lines. Loss of heterozygosity (LOH) was evaluated for *ACVR2* and *ACVR1*, and *ACVR2* promoter methylation by methylation-specific PCR and bisulfite sequencing and chromosomal instability (CIN) phenotype via fluorescent LOH analysis of 3 duplicate markers. *ACVR2* promoter methylation and ACVR2 expression were assessed in colon cancer cell lines via qPCR and IP-Western blots. Re-expression of ACVR2 after demethylation with 5-aza-2′-deoxycytidine (5-Aza) was determined. An additional 26 MSS colon cancers were assessed for ACVR2 loss and its mechanism, and ACVR2 loss in all tested cancers correlated with clinicopathological criteria.

**Results:**

Of 51 MSS colon tumors, 7(14%) lost ACVR2, 2 (4%) ACVR1, and 5(10%) pSMAD2 expression. No somatic *ACVR2* mutations were detected. Loss of ACVR2 expression was associated with LOH at *ACVR2* (p<0.001) and *ACVR2* promoter hypermethylation (p<0.05). *ACVR2* LOH, but not promoter hypermethylation, correlated with CIN status. In colon cancer cell lines with fully methylated *ACVR2* promoter, loss of *ACVR2* mRNA and protein expression was restored with 5-Aza treatment. Loss of ACVR2 was associated with an increase in primary colon cancer volume (p<0.05).

**Conclusions:**

Only a small percentage of MSS colon cancers lose expression of activin signaling members. ACVR2 loss occurs through LOH and *ACVR2* promoter hypermethylation, revealing distinct mechanisms for ACVR2 inactivation in both MSI and MSS subtypes of colon cancer.

## Introduction

Colon cancers with high frequency microsatellite instability (MSI-H) are associated with mutations in several genes with coding repetitive sequences, such as *transforming growth factor β receptor 2 (TGFBR2)* and *activin type 2 receptor (ACVR2)* [1]–[3]. Microsatellite stable (MSS) colon cancers have intact DNA mismatch repair, but may harbor *TGFBR2* kinase domain mutations [4]. Other components of the TGFβ canonical signaling cascade, such as SMAD2 and SMAD4, are specifically inactivated in a minority of colon cancers [5]–[7]. Systematic inactivation of TGFβ's sister pathway, activin, has not been fully elucidated in MSS colon cancers.

Activin is a member of the TGFβ superfamily that regulates cell differentiation in many tissues [8]. Similar to TGFβ, activin utilizes two cell surface receptors, activin receptor 1 (ACVR1) and activin receptor 2 (ACVR2), followed by SMAD activation. Another type 2 receptor, ACVR2B, cannot substitute for the functions and signaling of ACVR2 [9].

*ACVR2* was found mutated in the majority of MSI-H colorectal cancers [10], [11], primarily due to a frameshift in the A_8_ tract of exon 10. Restoration of activin signaling and growth suppression occur in response to *ACVR2* complementation in *ACVR2*-mutant colon cancer cells [12], [13]. We have previously demonstrated a high frequency of *ACVR2* mutations in MSI-H colon cancers in conjunction with loss of ACVR2 protein expression [2] and association with larger colon tumors and poorer histologic grade [14]. Also, we found a subset of MSS colon cancers that lost ACVR2 expression [2], akin to *TGFBR2* loss found in MSS colon cancers [4].

In this study, we explored activin signaling pathway disruption and possible mechanisms in primary MSS colon cancer specimens and colon cancer cells. We found that loss of ACVR2 expression occurs in a subset of MSS tumors, which is often associated with retained pSMAD2, the next downstream effector of both TGFβ and activin signaling. Unlike that of TGFBR2, ACVR2 loss in MSS tumors occurs through a combination of LOH at *ACVR2* and distinct *ACVR2* promoter methylation, but not genetic mutation. In colon cancer cell lines, mechanisms for ACVR2 loss also segregate according to microsatellite status, with MSI-H cell lines showing *ACVR2* polyadenine tract mutation and MSS colon cancer cells demonstrating promoter hypermethylation. Thus we show that disruption of activin signaling occurs in MSI and MSS colon cancers by distinct mechanisms, revealing activin signaling as an important target in the two most common genomic subtypes of colon cancer.

## Results

### Activin Signaling Pathway Members Are Targeted for Inactivation in Subsets of Primary MSS Colon Cancers

**Figure 1:**
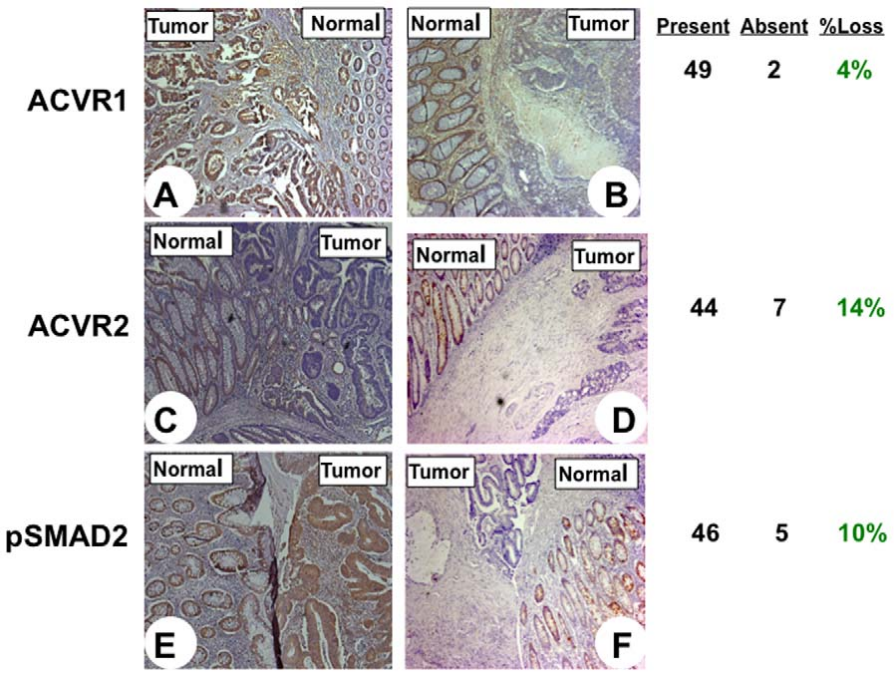
Loss of expression of three components of activin signaling in primary MSS colon cancers. Immunohistochemical analysis of paraffin-embedded primary colon cancers assessed expression of target protein (brown) as compared to adjacent normal tissue. A and B (Top row): ACVR1 expression is lost in a subset of MSS tumors. Example of tumor with expression in both normal and colon tumor tissue (left panel) and selective loss in a subset of tumors, but not adjacent normal colonic tissue (right panel). Overall, ACVR1 loss was observed in 2/51 or 4% of all MSS colon cancers analyzed. C and D (Middle row): ACVR2 expression is lost in a subset of MSS tumors. Two examples of selective loss of ACVR2 in colon tumor, but not normal colonic tissue (left and right panel) are shown. Overall, ACVR2 loss was observed in 7/51 or 14% of all MSS colon cancers analyzed. E and F (Bottom row): pSMAD2 expression is lost in a subset of MSS tumors. Example of expression in both normal and colon tumor tissue (left panel) and selective loss in a subset of tumors, but not normal colonic tissue (right panel). Overall, pSMAD2 loss was observed in 5/51 or 10% of all colon cancers analyzed.

Our previous data suggested at least partial loss of ACVR2 protein expression in a subset of primary MSS colon cancer specimens despite wild type *ACVR2* polyadenine tracts [2]. We examined this further and sought to determine expression patterns of both ACVR2, ACVR1 as well as its downstream effector, pSMAD2, in 51 different primary colon cancer specimens with microsatellite stable genomic backgrounds obtained from the same cohort of the North Carolina Colon Cancer Study (NCCCS) [15], [16]. While ACVR1 receptor expression was lost in only 4% (2/51) of the patient tumor specimens (**Figure 1AB, top row**), loss of the primary receptor, ACVR2, occurred in 14% (7/51) (**Figure 1CD, middle row**). Additionally, loss of pSMAD2 expression downstream of ACVR2 and ACVR1 activation by activin occurred in 10% (5/51) of tested cases (**Figure 1EF, bottom row**), and was commonly observed in tumors revealing fully expressed receptors (**Table 1**).

**Table 1:** Composition of protein expression of activin signaling proteins in colon cancers with loss of expression in at least one pathway member.

ID	ACVR1	ACVR2	TGFBR2	PSMAD2	SMAD2
MSS 884	absent	present	present	present	present
MSS 1026	present	absent	present	present	present
MSS 979	present	absent	present	present	present
MSS 1050	present	absent	present	present	present
MSS 1053	present	absent	present	present	present
MSS 796	present	absent	present	present	present
MSS 825	present	absent	present	present	present
MSS 1056	present	present	present	absent	present
MSS1052	present	present	present	absent	present
MSS 1004	present	present	present	absent	present
MSS 702	present	present	present	absent	present
MSS 994	absent	absent	present	absent	present

Two of the 51 tested MSS colon cancers lost ACVR1 protein expression, 7 lost ACVR2 expression, and 5 lost pSMAD2 expression. While one cancer lost expression of all three pathway components, the remaining 4 tumors with loss of pSMAD2 expression showed expression in activin's primary receptors, while maintaining expression of total SMAD2 and TGFBR2, indicative that loss of pSMAD2 may be a separate primary event. The remaining 39 primary MSS colon cancers revealed no loss of activin signaling components.

**Figure 2:**
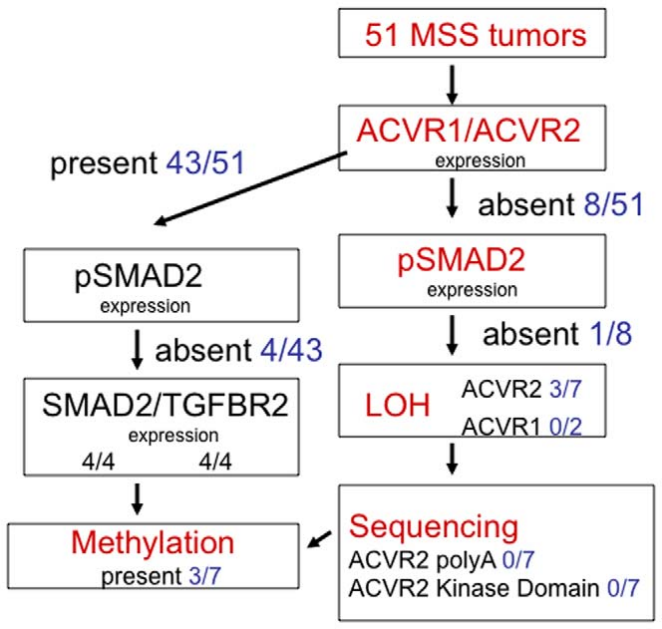
Summary of ACVR2 loss and its mechanisms in primary MSS colon cancer specimens. Of the 51 MSS colon cancers from the NCCCS cohort tested for ACVR2 and ACVR1 loss, 8 revealed loss of either receptor (7 lost ACVR2 and 2 lost ACVR1 with one tumor losing both, see Table 1). Of those 8, 1 lost pSMAD2. Of the 7 tumors with ACVR2 loss, 3 revealed LOH and 3 had selective ACVR2 promoter hypermethylation, while no mutations were found in any of the three kinase domain hotspots or the coding polyadenine tract of exon 10, commonly mutated in MSI colon tumors. Neither of the 2 tumors with ACVR1 loss revealed LOH at the *ACVR1* locus. Conversely, of the 43 tumors expressing both ACVR2 and ACVR1, 4 or 9% lost pSMAD2 expression, while maintaining total SMAD2 and TGFBR2 expression, underscoring loss of SMAD2 phosphorylation capability as an additional primary event to disrupt activin signaling.

All specimens with loss of at least one activin receptor pathway component revealed expression of both total SMAD2 and TGFBR2 (**Table 1** **,** **Figure 2**). These data suggest that ACVR2 protein loss has the greatest prevalence among MSS tumors, followed by pSMAD2 loss, and then by ACVR1 loss. ACVR2 loss and pSMAD2 loss appear to occur in complementary subgroups, suggesting more than one target to inactivating activin signaling in MSS colon cancers. Conversely, all MSS colon cancer specimens with activin signaling component loss expressed TGFBR2.

### LOH, but Not Mutation, Is Associated with Loss of ACVR2 Expression in Primary MSS Colon Cancer Specimens

To assess whether loss of receptor expression was due to loss of heterozygosity (LOH), a common genomic mechanism of tumor suppressor inactivation in MSS colon cancers, we assayed for LOH at the *ACVR2* and *ACVR1* gene loci. Of the 51 MSS tumors assayed, 4 revealed allelic loss. LOH at *ACVR2* was strongly associated with loss of ACVR2 protein expression (p = 0.006, Fisher's exact test), and 3/7 (43%) of MSS tumors with loss of ACVR2 protein expression also showed LOH (**Figure 2**), compared with 1/44 ACVR2 expressing tumors. No LOH was found at the *ACVR1* locus in either ACVR1 expressing or non-expressing tumors. For further ACVR2 expression correlation with LOH, we extended the number of patient tumors from 51 to 77 samples, which revealed 5 additional tumors with ACVR2 protein loss, bringing the total number to 12/77 or 16% (**Table 2**). In this expanded group, 6/12 (50%) of MSS tumors with ACVR2 loss showed LOH (**Table 2**).

**Table 2:** Mechanisms of *ACVR2* inactivation.

ID	ACVR2 Expression	ACVR2 LOH	ACVR2 Methylation	CIN
MSS 796	absent	no LOH	absent	yes
MSS 825	absent	no LOH	absent	yes
MSS 979	absent	no LOH	absent	yes
MSS 994	absent	no LOH	present	yes
MSS 1026	absent	no LOH	present	no
MSS 1053	absent	no LOH	present	no
MSS 747	absent	LOH	absent	yes
MSS 427	absent	LOH	absent	yes
MSS 345	absent	LOH	absent	yes
MSS 1050	absent	LOH	absent	yes
MSS 298	absent	LOH	absent	yes
MSS 325	absent	LOH	absent	yes

In the 12 total cancers with ACVR2 inactivation, 6 tumors revealed additional LOH at the *ACVR2* site, associated with CIN and 3 tumors revealed *ACVR2* promoter hypermethylation associated with LOH-/MSI- phenotype. No mutations in the kinase domain hotspots or coding polyadenine tract of *ACVR2* were found, implicating a combination of LOH and *ACVR2* promoter hypermethylation in ACVR2 expression loss. For three tumors, no mechanism for loss of *ACVR2* expression was identified.

We then sequenced the coding microsatellite as well as 3 separate hot spots in the kinase domain of *ACVR2* (akin to corresponding mutations within *TGFBR2* in MSS tumors)[4]. It should be noted that the exon 10 A_8_ tract in *ACVR2* lies in the kinase domain of this receptor, and frameshift mutations as well as other mutations in the kinase domain abolish the phosphorylating capacity of ACVR2. However, we found no tumor specific mutations corresponding with loss of ACVR2 protein expression. These data suggest that other inactivating mechanisms play a role in the loss of ACVR2 expression.

### *ACVR2* Promoter Hypermethylation Is Associated with Loss of ACVR2 Expression in Primary MSS Colon Cancer Specimens

**Figure 3:**
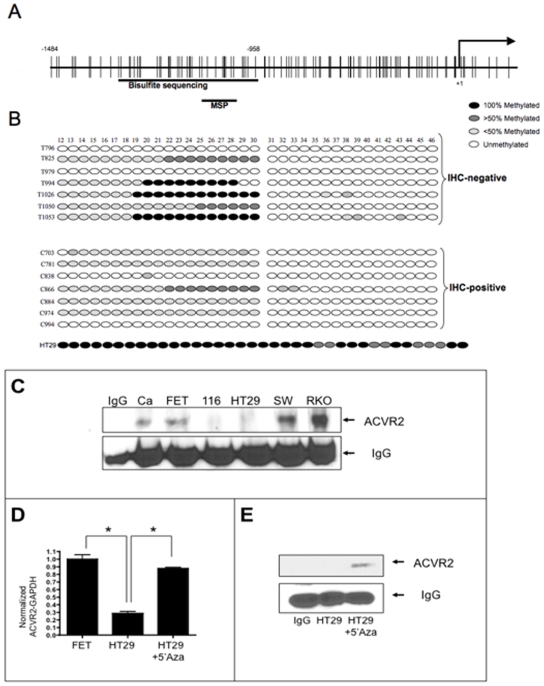
*ACVR2* promoter hypermethylation and LOH in colon cancer specimens and the MSS HT29 cell line and correlation of *ACVR2* promoter hypermethylation and loss of *ACVR2* expression. A) ACVR2 promoter with map of positioning of MSP primers B) Using a CpG islands search program, we identified the CpG islands within the *ACVR2* promoter based upon following stringent criteria: ∼CG percentage>55%; observed CpG/expected CpG >0.65; length >500 bp. All CpG dinucleotide sequences are represented by vertical bars across the horizontal line depicting the promoter sequence. Each circle in the bottom panel illustrates an individual CpG site corresponding to the vertical bars depicted in the upper panel. Based upon bisulfite sequencing, each CpG site was scored quantitatively as 100% methylated (dark circles), >50% methylated (dark grey circles), <50% methylated (light grey circles) or unmethylated (white circles). As indicated, 3 of 7 ACVR2-negative CRCs demonstrated complete methylation of the critical region of ACVR2 promoter, while none of the ACVR2-expressing tumors showed any evidence for high degree/complete methylation of ACVR2 promoter. A methylation pattern similar to that seen in ACVR2 negative colon cancers was observed in the MSS colon cancer cell line HT29 with genomic DNA from HT-29 allowing for sequencing of a slightly larger region of the *ACVR2* promoter. C) Six colon cancer cell lines with different microsatellite instability backgrounds (see Table 3) were analyzed for ACVR2 protein expression using immunoprecipitation techniques. Two cell lines, HCT116 (an MSI colon cancer cell line with biallelic frameshift mutations in *ACVR2*) and the MSS colon cancer cell line HT29 (with *ACVR2* promoter hypermethylation), revealed loss of ACVR2 protein expression. D) Loss of ACVR2 protein expression correlated with decrease in ACVR2 mRNA transcription via quantitative PCR in HT29 cells when compared to the ACVR2 expressing cell line FET using GAPDH for standardization. This experiment was performed three times in triplicates, and the bar graph represents one experiment with * indicating a statistically significant difference with a p<0.001. E) ACVR2 protein expression was re-established following demethylation treatment with 5′Aza.

To study whether epigenetic changes are associated with ACVR2 expression loss, we assessed whether the *ACVR2* promoter was hypermethylated in colon cancer tissue as compared to normal and if so, whether a specific methylation pattern of *ACVR2* correlated with ACVR2 protein loss in primary MSS colon cancer specimens. We initially divided the *ACVR2* promoter into three regions, region 1 (+142 to −603), region 2 (−607 to −958), and region 3 (−958 to −1484). Our bisulfite sequencing results showed that there was no methylation in region 1 and minimal methylation in region 2, but the 46 CpG dinucleotides between to −958 to −1484 nucleotides relative to the transcription start site (region 3) (**Figure 3A**) were methylated in both cell lines and clinical specimens (**Figure 3B**), but not corresponding adjacent normal tissue (data not shown). Due to the difficulty in amplification of larger amplicons from paraffin embedded tissue DNA, we performed methylation specific bisulfite sequencing of a slightly smaller region (−603 to −1297 positions) of the *ACVR2* promoter in the clinical specimens.

In the ACVR2 non-expressing primary colon cancers from the original sample size of 51 MSS tumors, 3/7 cancers demonstrated complete methylation (100% methylated alleles) within region 3 of the *ACVR2* promoter, while none of the 13 ACVR2 expressing primary colon cancers tissues assayed showed complete *ACVR2* methylation, supporting a causative role for methylation and lack of ACVR2 expression (p = 0.03, Fisher's exact test) (**Table 2**, **Figure 3B**). Bisulfite sequencing results were consistent with the MSP observations (data not shown), where 3/7 ACVR2-negative CRCs showed the presence of specifically methylated alleles. Although low levels of methylation (<50% methylated alleles) were also found in 7/13 of the ACVR2-expressing CRCs, none of the CRCs demonstrated complete methylation of any CpG dinucleotides, which was consistent with enabling ACVR2 expression in these tumors (**Figure 3B**).

### *ACVR2* Promoter Hypermethylation Correlates with ACVR2 Transcription and Protein Expression in Colon Cancer Cell Lines

To corroborate our findings from clinical specimens *in vitro*, we assessed mechanisms of *ACVR2* loss in colon cancer cells lines based on microsatellite instability. We tested 3 MSI-H and 3 MSS colon cancer cell lines for: 1) mutation in the coding polyadenine tract of exon 10 of *ACVR2*, 2) *ACVR2* promoter hypermethylation, 3) quantitative RT-PCR of *ACVR2* mRNA, and 4) ACVR2 protein expression by immunoprecipitation. As previously reported [2], [12], biallelic mutation in the polyadenine tract of *ACVR2* (A_8_ to A_7_) in the MSI-H colon cancer cell line HCT116 causes loss of ACVR2 protein (**Figure 3C** **and** **Table 3**).

**Table 3:** *ACVR2* expression and mechanisms for loss of expression in colon cancer cell lines.

	MSI Status	ACVR2 (WT = A8)	ACVR2 methylation	ACVR2 mRNA	ACVR2 protein
CaCo2	MSS	wildtype	partial	present	present
FET	MSS	wildtype	partial	present	present
HCT116	MSI	A7	partial	present	loss
HT29	MSS	wildtype	full	loss	loss
SW48	MSI	A7/8	partial	present	present
RKO	MSI	A6/8	partial	present	present

Using colon cancers cells with different microsatellite instability backgrounds, we confirm wild type A_8_ exon 10 polyadenine tract in all MSS colon cancer cell lines. As previously published, the MSI colon cancer cell line HTC116 harbors biallelic A_8_ to A_7_ frameshifts, leading to loss of full length ACVR2 protein [12]. Two MSI cell lines, SW48 and RKO, harbor mono-allelic mutations with no effect on ACVR2 gene expression. The MSS colon cancer cell line HT29 revealed full *ACVR2* promoter hypermethylation, akin to ACVR2 loss in primary human tumors, and was associated with loss of ACVR2 mRNA and protein.

In the MSI-H cell lines SW48 and RKO, ACVR2 protein is present, as it is in the MSS colon cancer cell lines CaCo2 and FET (**Figure 3C**). Both RKO and SW48 contain a heterozygous mutation at *ACVR2*, revealing wildtype A_8_ as well as mutant alleles (**Table 3**). The A_8_ *ACVR2* allele allows the expression of ACVR2 protein. The MSS colon cancer cell line HT29 expresses decreased levels of ACVR2 mRNA and protein (**Figure 3C, D and E**), unlike its MSS counterparts CaCo2 and FET, neither of which harbor any exonic *ACVR2* mutation (data not shown). HT29 cells revealed a distinct *ACVR2* promoter hypermethylation pattern (**Figure 3B**) in association with mRNA and protein loss, suggesting that this specific methylation pattern causes the loss of *ACVR2* expression. Demethylation of the *ACVR2* promoter with 5-Aza led to re-established expression of ACVR2 mRNA and protein (**Figure 3D and E**). We performed an additional screen of 11 colon cancer cell lines with either MSI (DLD1, HCA7, HCT15, LoVo, LS174, SNU175, SNU407), or MSS (SNU81, SNU503, SW480, and T84) backgrounds revealing similar levels of ACVR2 mRNA and establishing HT29 as the only observed MSS colon cancer model with distinct ACVR2 loss.

To correlate ACVR2 expression with tumor size, grade and stage, we analyzed the expanded group of 77 tumors, which contained 12 tumors with ACVR2 loss (**Table 2**). Of those, 69 had data on gender and race, 46 on tumor volume, 59 on tumor stage, and 65 on grade (see Table 4) allowing subanalyses. Akin to MSI-H colon cancers [14], loss of ACVR2 expression correlated with larger tumors (p = 0.024), while stage was unaffected when compared to ACVR2-expressing tumors (Table 4). This parallels our previous finding in MSI-H colon cancers, where loss of ACVR2 protein was associated with larger, more poorly differentiated tumors in a stage-independent fashion [14].

**Table 4:** Loss of *ACVR2* expression is associated with larger tumor volume.

	ACVR2 Expression	Loss of ACVR2 Expression	*P*-value
Age, mean	63.60	61.82	0.620
Gender			
Male, n, (%)	31/58 (53)	4/11 (36)	0.299
Female, n, (%)	27/58 (47)	7/11 (64)	
Race			
White, n, (%)	22/58 (38)	7/11 (64)	0.113
Black, n, (%)	36/58 (62)	4/11 (36)	
Tumor Volume	n = 40	n = 6	
Mean	16.11	35.73	0.024*
Median	8.70	28.73	
Tumor Stage	n = 49	n = 10	
Duke A & B, n, (%)	41/49 (84)	10/10 (100)	0.169
Duke C & D, n, (%)	8/49 (16)	0/10 (0)	
Grade	n = 55	n = 10	
Well & ModeratelyDifferentiated, n, (%)	50/55 (91)	8/10 (80)	0.306
PoorlyDifferentiated, n, (%)	5/55 (9)	2/10 (20)	

ACVR2-expressing and non-expressing cancers were assessed for correlation of ACVR2 status with age, gender, race, tumor volume, stage and a grade. * p<0.05.

We then performed genomic subtype analysis of all ACVR2 non–expressing cancers and found that the lack of the chromosomal instability (CIN) phenotype correlated with *ACVR2* promoter hypermethylation (Table 2), suggesting separate pathways for MSI-/LOH+ and MSI-/LOH- colon cancers.

Taken together, these data suggest that the clinico-pathologic effects of ACVR2 protein loss may be similar in both MSI and MSS colorectal cancers despite differing underlying mechanisms of loss, implicating ACVR2 loss as an important step in colon carcinogenesis.

## Discussion

Disruption of activin signaling is common in MSI-H colon cancer cell lines through mutation of *ACVR2* in one of its polyadenine tracts [10], causing loss of ACVR2 protein [2]. Loss of ACVR2 protein expression was also noted in a small subset of MSS colon cancers [2]. Here, we assessed the occurrence and mechanism of disrupted activin signaling in MSS colon cancers and demonstrate that activin signaling is targeted for disruption at multiple levels in MSS colon tumors. Most commonly, *ACVR2* expression is lost via a combination of LOH and epigenetic silencing of the *ACVR2* promoter. These findings underscore the importance of abrogated activin signaling in colon tumorigenesis, as its disruption occurs in both MSI and MSS subtypes of colon cancer by differing distinct mechanisms.

In MSI-H colon cancers, both TGFβ and activin are abrogated due to frameshift mutations in the type II receptor [2], [17]. The loss of both of these signaling pathways may be beneficial for tumor growth [12], [18]. Both TGFβ and activin use the same intracellular SMAD proteins (SMAD 2 and 3) to transmit their signal. We previously observed greater than 50% overlap between *ACVR2* and *TGFBR2* mutations in primary MSI colon tumors [2], possibly because of additive effects in mediating the growth response, which are currently under investigation.

It appears that in MSI colon cancers *ACVR2* mutations may occur early in tumorigenesis and are associated with increased local growth [14]. In MSS colon cancers, loss of ACVR2 correlated with larger tumors, consistent with disruption of activin-induced growth suppression. The timing of *ACVR2* mutations in MSI colon cancer may be similar to that of *TGFBR2* in which the frameshift mutations occur in high grade dysplasia at the interface to malignancy [17].

We show that in MSS colon cancers at least three members of the activin signaling cascade, ACVR2, ACVR1, and pSMAD2 are disrupted. A significant subset of colon tumors displayed a decrease in phosphorylated SMAD with intact ACVR2 and TGFBR2, indicating a separate primary event downstream of the primary receptors. One case of loss of ACVR1, ACVR2 and pSMAD2 was identified, which was TGFBR2 staining positive (Table 2). This could be due to primary inactivation of pSMAD2 and separate targeting of both activin receptors, or an IHC positive truncated TGFBR2, leading pSMAD2 loss. The effect of loss of multiple targets of the same signaling cascade still needs to be carefully explored and suggests distinct functions of each member.

We detected LOH at the *ACVR2* locus in 6% of MSS colon tumors, increasing to 50% in tumors with loss of ACVR2 protein expression. This overall rate is slightly lower than the frequency of *ACVR2* LOH reported previously in a cohort with unknown *ACVR2* status [19], although we have previously observed cohort-dependent frequencies for ACVR2 expression that may be stage or race-dependent [14].

Our mutational analysis focused on hotspots akin to those implicated in inactivation of the *TGFBR2* kinase domain inactivation [4], but we did not find any tumor-associated mutations when sequencing all exons in ACVR2 expressing and non-expressing colon cancer cell lines. It is possible that mutations outside of the hotspots may contribute to loss of ACVR2 in primary colon cancer specimens, although in light of the evidence for LOH as well as epigenetic silencing, this is likely to be a less important tumorigenic mechanism. However, it may play a role in the three tumors with loss of ACVR2 where we found no genetic or epigenetic mechanism directly explaining that loss.

This is the first report describing ACVR2 loss in MSS colon cancers and *ACVR2* promoter hypermethylation as a mechanism, and contrasts with the mechanisms of *ACVR2* loss in the MSI colon cancer cells [12]. In both primary MSS colon tumors and in HT29 colon cancer cells, the region of promoter hypermethylation that is associated with loss of *ACVR2* expression is between nucleotides −1297 to −958. Among genes that are targets for epigenetic silencing, *hMLH1* is the best studied for methylation, and it has been proposed that the promoter region adjacent to the transcription start site (TSS) is critical for transcriptional silencing of this gene [20]. There is lack of similar data for most other genes, but *hMLH1* promoter region data is often used as a paradigm, and it is believed that for most genes, analysis of a similar promoter region is critical to correlate DNA methylation findings with loss of gene expression. In this study, we have analyzed >1500 bp proximal to the TSS, and found good correlation between *ACVR2* promoter methylation (in regions −958 to −1297) and loss of protein expression by IHC. Our data suggests that instead of mere proximity to the TSS, differential access to methylation co-activators or repressors in a promoter determines gene silencing, and for *ACVR2* such a region may occur upwards of 900 bp from the TSS.

We are mindful that the total number of MSS patients with ACVR2 loss that were investigated in this study is not large, underscoring that this is a relatively infrequent event [15]. We did not intend to determine the overall frequencies of such events, but to show an alternative mechanism of ACVR2 protein loss in MSS colon cancers. Of 51 primary, population-based MSS tumor samples, 7 showed loss of ACVR2 expression. In keeping with the 51 subjects being randomly drawn from the cohort, between 7% and 21% of MSS tumors in the population should show similar loss of ACVR2 expression with 95% confidence (Clopper-Pearson exact confidence interval). Increasing the sample size to 77 revealed loss of ACVR2 in 12/77 or 16% and a statistically significant correlation of loss of ACVR2 with increased tumor size. Further, genomic subtype analysis revealed that LOH at *ACVR2* was associated with the CIN phenotype, while *ACVR2* hypermethylation correlated with the CIMP phenotype. While these categorizations allow attribution of loss of *ACVR2* expression to promoter hypermethylation and/or chromosomal instability as a mechanism in MSS cancers, alternative mechanisms such as histone modification and/or microRNAs may be at play, particularly in the LOH negative/methylation negative cancers. Our data however, show a significant correlation between loss of ACVR2 expression and LOH/epigenetic silencing. Thus we provide evidence for the existence of either chromosomal instability or epigenetic modification of *ACVR2* in colon cancer and identify a cell model for epigenetic silencing of *ACVR2*. The full clinical impact of this data will require further confirmation in future studies with larger patient samples.

In conclusion, loss of ACVR2, ACVR1 and pSMAD2 expression occurs in a subset of MSS tumors, and the evidence supports that this results in abrogation of the normal growth suppressive activity of activin signaling. Decreased pSMAD2 is commonly associated with wild type ACVR2 and ACVR1. Mechanisms for *ACVR2* loss include LOH at *ACVR2* and *ACVR2* promoter hypermethylation between nucleotides −1297 and −958, which are associated with CIN and CIMP phenotypes, respectively. Loss of ACVR2 is associated with increased tumor size. Therefore, activin signaling can be inactivated by distinctive mechanisms in MSI and MSS colon cancers, suggesting the importance of this pathway in controlling colonocyte growth.

## Materials and Methods

### Ethics Statement

This study was conducted according to the principles expressed in the Declaration of Helsinki. The study was approved by the Institutional Review Board of the University of North Carolina hospitals. All patients provided written informed consent for the collection of samples as part of the under IRB approval as part of the North Carolina Colorectal Cancer Study (NCCCS) see below. Analysis as part of this study was completely de-identified and no identifying information was available to the PI, making this study exempt from a separate consent.

### Patient Samples

Sporadic colon tumors were prospectively collected under IRB approval as part of the North Carolina Colorectal Cancer Study (NCCCS), a population-based, case-control study comprising 503 patients [15], [16]. Microsatellite analysis was performed from paraffin-embedded tissue as previously described [2], segregating the cohort into 54 MSI-H, and 449 MSS/MSI-L patients. For this study, 51 MSS patient samples with ample tumor and normal tissue were randomly selected.

### Cell Lines

The MSI colon cancer cells lines HCT116, SW48, DLD1, HCA7, HCT15, LoVo, LS174T, SNU175, SNU407, SW48, and RKO, as well as the MSS colon cancer cell lines CaCo2, HT29, SNU81, SNU503, SW480, and T84 were maintained in Iscove's Modified Dulbecco's Medium (Invitrogen Corporation, Carlsbad, CA) and FET were maintained in F12/Dulbeco's Modified Eagles Medium at conditions previously described [12]. All cell lines are available from ATCC except for FET (kind gift of Michael Brattain, Medical College of Ohio, Toledo, OH) [21].

### Tissue Microdissection, DNA Extraction, and RNA Extraction

DNA from the formalin-fixed, paraffin-embedded material was extracted following microdissection using the Takara DEXPAT kit (Takara Bio Inc., Japan). Genomic DNA from cells lines was obtained using QIAamp DNA mini kit (Qiagen, Valencia, CA). RNA from cell lines was obtained using the TRIzol reagent (Life Technologies Inc. Carlsbad, CA).

### Antibodies

Rabbit anti-TyrGly mACVR2 (482–494) (generous gift from W. Vale, Salk Institute), with its target epitope in the C-terminus region of ACVR2 (beyond the resulting truncation from frameshift in exon 10) was used for immunohistochemistry as previously described [2], [12]; rabbit anti-TyrGly ACVR1 (474–494) was used at 1∶800; pSMAD2 (Cell Signaling) at 1∶250; total SMAD2 (Epitomics) at 1∶400; and TGBR2–C16 (Santa Cruz) at 1∶300.

### Immunohistochemistry for ACVR1, ACVR2, pSMAD2, TGFBR2 and SMAD2 Protein Expression

Immunohistochemistry was performed as described previously [2]. Staining was grouped into loss or 0 (absence or significant decrease as compared to adjacent normal tissue), reduced or 1+ (subtle decrease as compared to adjacent normal tissue), unchanged or 2+ (no change as compared to adjacent normal tissue), and increased or 3+ (increase as compared to adjacent normal tissue). Three independent investigators blindly scored all slides. All three investigators had to be in agreement for a tumor to be called 0 or loss of expression.

### Loss of Heterozygosity (LOH) Analysis

Assessment of LOH at the *ACVR2* and *ACVR1* loci was performed as previously described [22] utilizing 2 microsatellite markers (D2S1353 and D2S1399) flanking *ACVR2* [19] as well as an intragenic marker for *ACVR1* (D2S2686) (see **Table S1**).

### CIN Analysis

Analysis for CIN was performed as previously described [23]. Briefly, forward oligonucleotide primers were fluorescent-labeled with FAM at the 5′end (Applied Biosystems) and a set of 6 polymorphic microsatellite markers (D5S346, D5S409 D17S261 D17S250 D18S81, D18S91, and D18S69) (see **Table S1**) was used to determine LOH at chromosome 5q, 17q and 18q. PCR amplifications were performed on genomic DNA templates from both tumor and corresponding normal tissues. The amplified fluorescent PCR products were electrophoresed on an ABI PRISM 3100 Avant Genetic analyzer and analyzed by GeneMapper fragment analysis software (Applied Biosystems). When comparing the signal intensities of individual markers in tumor DNA with that of the corresponding normal DNA, a reduction of at least 40% in the signal intensity was considered indicative of LOH.

### Bisulfite Modification, Methylation Specific PCR (MSP) and Bisulfite Sequencing

DNA from matched colon cancer tissues and corresponding normal mucosa from CRC patients as well as DNA from colon cancer cell lines underwent bisulfite modification as described previously [24].

For methylation analysis, we first identified the tentative CpG islands in the promoter region of the *ACVR2* gene based upon mapping analysis using CpG island search program (http://www.uscnorris.com/cpgislands/cpg.cgi). Accordingly, we designed multiple primer sets for MSP and bisulfite sequencing that spanned the entire *ACVR2* promoter region, including part of exon 1, to determine methylation density across all CpG dinucleotides with methylation specific primers (see **Table S1**). Bisulfite sequencing was initially performed on colon cancer cell lines as it allowed amplification of larger amplicons, and helped determine critical regions of promoter methylation that were subsequently PCR amplified in the clinical samples.

### ACVR2 Genotyping

Three hotspots in conserved regions of the kinase domain of *ACVR2* [25], homologous to the previously identified point mutations in *TGFBR2* [4] as well as the coding A_8_ microsatellites of *ACVR2* in exon 10 [2], were amplified using specific primers (see **Table S1**) and followed by sequencing as previously described [2]. All exons of *ACVR2* were amplified from genomic DNA extracted from select ACVR2 expressing and non-expressing colon cancer cell lines using specific primers (see **Table S1**) and subjected to sequencing using the DNA Sequencing Shared Resource, UCSD Cancer Center. Any new mutations were to be deposited to GenBank.

### Quantitative Expression of ACVR2 mRNA

To detect the amplification of *ACVR2* in colon cancer cells, we performed a quantitative polymerase-chain reaction. Briefly, all 17 colon cancer cell lines were grown to 60% confluence. RNA was extracted using a Trizol-based protocol. The concentration of RNA dissolved in DEPC-treated water was assessed using a Beckman-Coulter DU640B spectrophotometer (Beckman-Coulter, Fullerton, CA). We performed RT-PCR using an oligo dT for incubation at 37°C to generate cDNA as previously described [12]. Then, quantitative PCR was carried out using specific primers for exon 10 (see **Table S1**) Templates from each cell line were prepared in triplicate per target gene as 10 µL reactions (40 ng template, 2X Mesa Green qPCR MasterMix for SYBR assay, 100X forward and reverse primers). Templates were plated on fast optical 96-well plate (Applied Biosystems, Foster City, CA) and spun for 2 minutes at 2000 rpm. Results were observed and analyzed on the StepOnePlus Real-Time PCR System (Applied Biosystems, Foster City, CA). GAPDH mRNA amplification was performed in parallel (5′- CATGTTCGTCATGGGTGTGAACCA-3′, 5′-AGTGATGGCATGGACTGTGGTCAT-3′) to obtain a normalized ACVR2-GAPDH after the relative expression of each gene was calculated using standard curves.

### Immunoprecipitation (IP)

To detect presence of ACVR2 in the colon cancer cell lines, we performed immunoprecipitation with ACVR2 followed by Western blotting as previously described [12]. Then, a subset of HT29 cells was either treated with the demethylating agent 5-Aza (Sigma) for 72 hours or vehicle prior to lysis and assessment of ACVR2 expression.

### Statistical Analysis

Analysis was performed with the help of the UCSD Moores Cancer Center Biostatistics Core (K.M.) applying Fisher's exact test and Student's t-test with a p value of <0.05 indicating statistical significance. Further, Clopper-Pearson exact confidence interval was used to determine the 95% confidence interval.

## Supporting Information

Table S1 Specific primers used in LOH analysis, ACVR2 genotyping as well as ACVR2 promoter bisulfite sequencing. F denotes forward primer, R denotes reverse primer; U denotes unmethylated and M methylated.
(0.09 MB DOC)
