# Supplementary material for: Activin Signaling in Microsatellite Stable Colon Cancers Is Disrupted by a Combination of Genetic and Epigenetic Mechanisms
Source: PLoS One. 2009 Dec 14;4(12):e8308. doi: 10.1371/journal.pone.0008308 (PMC2789408; doi:10.1371/journal.pone.0008308)
Supplement: Table S1 — Specific primers used in LOH analysis, ACVR2 genotyping as well as ACVR2 promoter bisulfite sequencing. F denotes forward primer, R denotes reverse primer; U denotes unmethylated and M methylated. (0.09 MB DOC) [file pone.0008308.s001.doc]

**SUPPLEMENTAL DATA**

| Primer name | Sequence |
| --- | --- |
| D2S1353-F | 5’-CCAGGGACATTGCTTAACAT-3’ |
| D2S1353-R | 5’-GAGCAGGATTTGTAACCCTG-3’ |
| D2S1399-F | 5’-CATTGGTCCAGGTAAACTGC-3’ |
| D2S1399-R | 5’-TTCACAAGGTTCCACAAGGT-3’ |
| D2S2686-F | 5’-AAAGGCAGCCAAATCTAATAACA-3’ |
| D2S2686-R | 5’-TCACTCTACCAAAGATTTGGCA-3’ |
| ACVR2 coding polyadenine-F | 5’-GTTGCCATTTGAGGAGGAAA-3’ |
| ACVR2 coding polyadenine-F | 5’-GCATGTTTCTGCCAATAATCTC-3’ |
| ACVR2 hotspot 1-F | 5’-ACCCCCACCTTCTCCATTAC-3’ |
| ACVR2 hotspot 1-R | 5’-CCACCTTGGTACATATGGCTT-3’ |
| ACVR2 hotspot 2-F | 5’-TTTGCAAGGGACATCAAAAG-3’ |
| ACVR2 hotspot 2-R | 5’-TTTGCAAGGGACATCAAAG-3’ |
| ACVR2 hotspot 3-F | 5’-GACCACATTTGGTTTTGATTC-3’ |
| ACVR2 hotspot 3-R | 5’-CGAGAAGCCAGTTCCCACTAG-3’ |
| ACVR2 exon1-F | 5’-ACACCAGGAGGTTTGTCTCC-3’ |
| ACVR2 exon1-R | 5’-GGACTCTCCGACTCAACACC-3’ |
| ACVR2 exon2-F | 5’-GGGAAGACGGTGAATTACTGA-3’ |
| ACVR2 exon2-R | 5’-TCAAAAAGAGGGGAAGTGTGA-3’ |
| ACVR2 exon3-F | 5’-AAAAACACTTGTTGTAGGGTCAG-3’ |
| ACVR2 exon3-R | 5’-TGTTTCCAATCTACAGTTGAGCA-3’ |
| ACVR2 exon4-F | 5’-AGACCAAATCTGAGTTATTTTTCC-3’ |
| ACVR2 exon4-R | 5’-CAATTTAAAACACTACAAAGTACAGGA-3’ |
| ACVR2 exon5-F | 5’-TGGTGTGTGTCATGTTCTGCT-3’ |
| ACVR2 exon5-R | 5’-TGTATGTAAGATGACAAAACTGCAA-3’ |
| ACVR2 exon6-F | 5’-TTAAACCTGTATTCCTTGTGTTCTT-3’ |
| ACVR2 exon6-R | 5’-TAGGCAGGCCAACTCAGACT-3’ |
| ACVR2 exon7-F | 5’-CATATGGCCTTTGTCAAGAACA-3’ |
| ACVR2 exon7-R | 5’-CTCAATTATCTGGGAAAACAATATAA-3’ |
| ACVR2 exon8-F | 5’-CCCCCTTTTCTGCTTTCAAT-3’ |
| ACVR2 exon8-R | 5’-TTCAGTCCTGACATGCCATT-3’ |
| ACVR2 exon9-F | 5’-TGAGTACTCTTTGCTTTTAACATCTTT-3’ |
| ACVR2 exon9-R | 5’-TGGAGTGTATCATCCCTTTTCAT-3’ |
| ACVR2 exon10-F | 5’-CCAGTTTGAAAGTCAGGAGGA-3’ |
| ACVR2 exon10-R | 5’-TGGAATTTCAAATGAAAAGCTAAC-3’ |
| ACVR2 exon11-F | 5’-CTGCTGTGGCGTTTGAGTAT-3’ |
| ACVR2 exon11-R | 5’-GCAGCTCCAGTTCAGAGTCC-3’ |
| ACVR2A-U-F | 5’-AGTTAGTTTTTAGATTGTTTGGGT-3’ |
| ACVR2A-U-R | 5’-AAATCACTATCAACTCCACTCA-3 |
| ACVR2A-M-F | 5’-GTTAGTTTTTAGATTGTTCGGGC-3’ |
| ACVR2A-M-R | AAATCACTATCAACTCCGCTCG |
| MSP_UF | 5’-AGTTGTTGTAAAGTTGGTGTTTGTTG-3’ |
| MSP_UR | 5’-AAACTCTCCAACTCAACACCAACCA-3’ |
| MSP_MF | 5’-AGTTGTTGTAAAGTTGGCGTTTGTC-3’ |
| MSP_MR | 5’-AAACTCTCCGACTCAACACCAACCG-3’ |
| ACVR2A_BIS_BF | 5’-TTTTTTTTGGGTTTTTTTTGATTT-3’ |
| ACVR2A_BIS_BR | 5’-TAATCCAAACTCTAACTCCAACTCC-3’ |
| D5S3465q-F | 5’-6FAMAGCAGATAAGACAGTATTACTAGTT-3’ |
| D5S3465q-R | 5’-ACTCACTCTAGTGATAAATCGGG-3’ |
| D5S4095q-F | 5’-6FAMGGGATGAAGTGTGGATAAACT-3’ |
| D5S4095q-R | 5’-AGGATGGCAGTGCTCTTAG-3’ |
| D17S261-F | 5’-6FAMAGGGATACTATTCAGCCCGAGGTG-3’ |
| D17S261-R | 5’-ACTGCCACTCCTTGCCCCATTC-3’ |
| D17S250-F | 5’-6FAMGGAAGAATCAAATAGACAAT-3’ |
| D17S250-R | 5’- GCTGGCCATATATATATTTAAACC-3’ |
| D18S691-F | 5’-6FAMAATCCTCTTTCTCTGACTCTGA-3’ |
| D18S691-R | 5’- ATGTTCCCCGCTATTGTACT-3’ |
| D18S681-F | 5’-6FAMGCACCAACAGTGTCCACTATAC-3’ |
| D18S681-R | 5’- GATGCTGCTGGTCTGAGG-3’ |

**Supplemental Table 1.** Specific primers used in LOH/CIN analysis, *ACVR2* genotyping as well as *ACVR2* promoter MSP and bisulfite sequencing. F denotes forward primer, R denotes reverse primer; U denotes unmethylated and M methylated.
